# Novel 1, 4-dihydropyridines for L-type calcium channel as antagonists for cadmium toxicity

**DOI:** 10.1038/srep45211

**Published:** 2017-03-27

**Authors:** Madhu Sudhana Saddala, Ramesh Kandimalla, Pradeepkiran Jangampalli Adi, Sainath Sri Bhashyam, Usha Rani Asupatri

**Affiliations:** 1 Division of Bioinformatics, Department of Zoology, Sri Venkateswara University, Tirupati, 517502, A.P., India; 2 Garrison Institute on Aging, Texas Tech University of Health Science Centre, Lubbock, USA; 3 CIMAR/CIIMAR, Centro Interdisciplinar de Investigação Marinha e Ambiental, Universidade do Porto, Rua dos Bragas, 177, 4050-123 Porto, Portugal; 4 Vikrama Simhapuri University, Nellore-524 003, Andhra Pradesh, India

## Abstract

The present study, we design and synthesize the novel dihydropyridine derivatives, i.e., 3 (a-e) and 5 (a-e) and evaluated, anticonvulsant activity. Initially due to the lacuna of LCC, we modeled the protein through modeller 9.15v and evaluated through servers. Docking studies were performed with the synthesized compounds and resulted two best compounds, i.e., **5a, 5e** showed the best binding energies. The activity of intracellular Ca^2+^ measurements was performed on two cell lines: A7r5 (rat aortic smooth muscle cells) and SH-SY5Y (human neuroblastoma cells). The 5a and 5e compounds was showing the more specific activity on L-type calcium channels, i.e. A7r5 (IC_50_ = 0.18 ± 0.02 and 0.25 ± 0.63 μg/ml, respectively) (containing only L-type channels) than SH-SY5Y (i.e. both L-type and T-type channels) (IC_50_ = 8 ± 0.23 and 10 ± 0.18 μg/ml, respectively) with intracellular calcium mobility similar to amlodipine. Finally, both *in silico* and *in vitro* results exploring two derivatives 5a and 5e succeeded to treat cadmium toxicity.

Channels are the building blocks for many metabolic regulations and act as check boundaries for the entry of many nutrients and minerals. Toxic metals may also enter through these channels and causes adverse metabolic regulations thereby leading to cell poison. The Entry of heavy metals like cadmium through channel plays the crucial role and key finding in cadmium toxicity. Present scenario, the understanding the cadmium entry through channels and targeting these channels with drug discovery process are very attractive and novel to treat the toxicity. Cadmium (Cd) is an extremely toxic metal commonly found in industrial workplaces. It is also a food contaminant and a major component of cigarette smoke. Cd can enter the brain parenchyma and neurons, causing neurological alterations in humans and animal models, leading to lower attention, hypernociception, olfactory dysfunction, and memory deficits^1^.

Cd influx mediates voltage-gated calcium channels (VDCCS) in excitable cells^2^ including mammalian neurons and also Cd uptake in non-excitable tissues^3^. They are transmembrane proteins, which are playing an integral role in the entry of Ca^2+^ permeation in excitable cells and also in controlling synaptic transmission, muscle contraction, gene transcription, cell division, cell death, hormone (or) Neuro transmitter and signal transduction pathways^4^. L-type Ca^2+^ channels (LCCs) are multi subunit complex and heteromeric proteins consisting of the pore forming appha-1 (α1) subunit, disulfide-linked transmembrane complex of alpha-2 (α2), intra cellular beta (β) subunit, delta (Δ) and a gamma (γ) subunit characteristics of skeletal muscle Ca^2+^ channels in a 1:1:1:1 ratio^5^,^6^. The pore forming α1 subunit directs the channel activity that displays the major pharmacological and electrophysiological properties. The pore formingα1 subunit of LCCs folds from a single polypeptide chain, composed of four distinct repeats (I-IV), each repeat formed by six transmembrane segments (S1-S6). S1-S4segments have a voltage sensing domain, an outer helix S5, an inner helix S6 and a membrane dividing P-loop between S5-S6. The EEEE ring (selecting filter glutamates) in four repeats (I-IV) of LCC^5^,^6^. This ring in P-loops forms the selectivity filter for metal ions. Calcium channel blockers proceed onion conducting cell membrane channels.

The 1,4-dihydropyridine (DHP) class of calcium channel blockers are widely utilized in the treatment of cardiovascular diseases such as hypertension, angina pectoris, and other spastic smooth muscle disorders^1^. The SAR of calcium channel blockers signifies the presence of ester linkage and electron withdrawing groups like nitro and carbonyl groups. The Pyrimidine nucleus has been showing the various pharmacological activities like calcium channel blockers, anti-cancer, antimicrobial, antiviral. Calcium channel blockers proceed onion conducting cell membrane channels. The1,4-dihydropyridine moiety is commonly useful as calcium channel blockers and are used most frequently as drugs such as nifedipine, diltiazem, nicardipine, amlodipine (AD), which have been found as potent cardiovascular agents for the treatment of hypertension. Hence this class of agents may be included in the search for protectors with a more favorable therapeutic index. Therefore, in the present study, an attempt has been made to find out the detoxifying action of calcium channel blockers on L-type calcium channel against Cd toxicity through synthesis the 1,4-dihydropyridine derivatives of compounds and test the *in silico* and *in vitro* approaches to find the new therapeutics against Cd toxicity.

## Results and Discussion

### Sequence alignment

The homology modeling study is the most important for proper alignment of the sequence of the target protein with that of the template protein. The voltage-gated LCC share structural similarities with K and Na channels in that they possess a pore-forming *α*1 subunit in four repeats of a domain with six transmembrane-spanning segments that include the voltage-sensing S4 segment and the pore-forming (P) region. There is no atomic resolution images of calcium channel structures exist, much has been learned about their structure since the recent determination of crystal structures of a number of potassium channels^6^,^7^. The *α*1 subunit contains four repeated domains (I–IV), each of which includes six transmembrane segments (S1–S6) and a membrane associated loop (the “P-loop”) between segments S5 and S6. The four repetitive domains are in addition; extraordinarily comparable to those recognized to form the voltage-gated potassium channels. However, potassium *α*1 subunit is homotetramer and calcium channel is heterotetramer. Potassium channel KcsA (PDB: 1BL8) has been selected to be the template. Amino acid sequences of S5, S6, and P-loops in between for the four repeats (I–IV) (271–405, 654–753, 1052–1185 and 1411–1524, respectively) of voltage-dependent LCC subunit alpha-1C (CAC1C HUMAN Q13936) were used for sequence alignment with the amino acid sequence of KcsA as proposed by Zhorov *et al*.^8^, where S6 segments of LCC are aligned with M2 segments of KcsA in a manner similar to the alignment of the Na channel with KcsA described by *Lipkind and Fozzard* (2000)^9^ and S5s were aligned with the M1 segment of KcsA as proposed by Huber *et al*.^10^ and the P loops were aligned using MULTALIN server^6^,^11^ (Supporting Information Figure S1). Proteins that wrinkle into like structures can have big differences in the range and form of residues at corresponding positions. These changes are tolerated not only because of replacements or movements in nearby side chains, as explored by Ponder and Richards, but also as a result of shifts in the backbone^6^. For an additional flexible inspection, the sequence of each replicate was prepared as S5, S6, and P-loop, allowing easier corrections. The amino acid sequence of repeats I and III have a long extracellular loop which would reduce the quality of the generated model, so amino acid sequences were excluded from repeats I and III, respectively. Since the template is 88 residues shorter than the target protein, gaps were inserted to attain finest sequence comparison and distinctiveness without disturbing sequence arrangement planned by Zhorov *et al*.^8^. The furthermost consideration was thus paid to the watchful construction of transmembrane helices S5 and S6 and P-loop as well.

### Construction of the LCC Model

Although the two proteins have a low sequence identity of 9.5% and sequence comparison of 29.2%, the Modeller program was practical to produce agreeable models. As an essential progression of model building, original refinement of the loop conformation after model generation was automatically performed by Modeller Loop refinement-DOPE-Loop method during the process. The model developed from the alignments by Zhorov *et al*.^8^ was subjected to extensive loop optimization. This procedure is based on the idea that transmembrane helices are much less flexible than loops, thus permitting to construct a sounder core arrangement if the reliability of the helices is conserved. On the divergent, the more unpredictable loops can bear the most significant distinction connecting the coordinates of the reference and the model. While a homology model is produced, there are parts of the model sequence which are not aligned to any template structures. For these sections, no homology restraints (such as C*α*-C*α* distance restraints) can be applied. These parts of the structure commonly have superior errors compared to the regions which are modeled based on a template structure. In an attempt to condense these errors, a CHARMM-based procedure that optimizes the conformation of a contiguous segment (i.e., a loop) of a protein structure called loop refinement was applied^12^. This is based on the methodical conformational example of the loop backbone and CHARMM energy minimization. The algorithm goes all the way through three stages: construction and optimization of loop backbone, construction of loop, side-chain and optimization of loop followed by re-ranking of the conformations. The model was then tartan after a methodical energy minimization considered diminishing the steric clashes of the side chains without modifying the backbone of the protein to resolve these contacts. To avoid adjustment of the backbone of the protein, the optimization of the geometry of side chain was performed with constraining the backbone. After the optimization, models were tartan to evaluate the superiority of their structure.

### Model Evaluation

**Figure 1:**
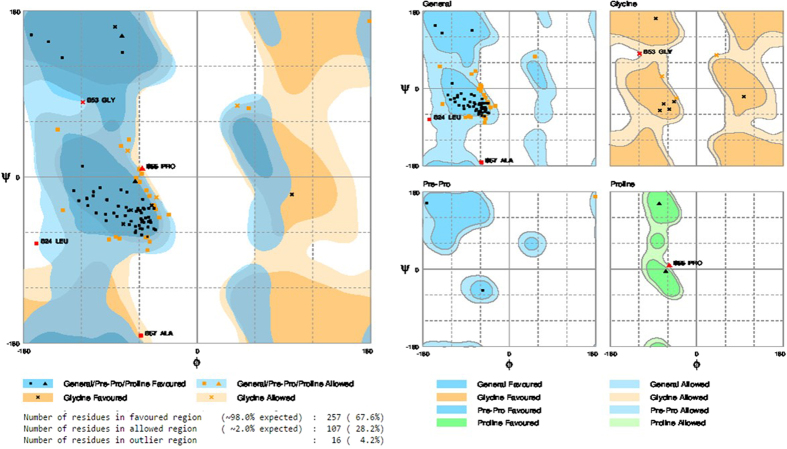
Rampage analysis of Ramachandran plot in order to visualize energetically allowed regions for backbone dihedral angles ψ against φ of amino acid residues in modelled protein structure (LCC model). (**A**) The plot of LCC model shows that number of residues found in the favored region 98.0% from the total residues, and number of residues in allowed region is 2.0% and number of residues in outlier region 0.0%. (**B**) The LCC model shows general, glycine, pre-proline, and proline plots exhibits the possible ψ and φ dihedral angles and allowed and disallowed regions of complete amino acids of LCC.

**Figure 2:**
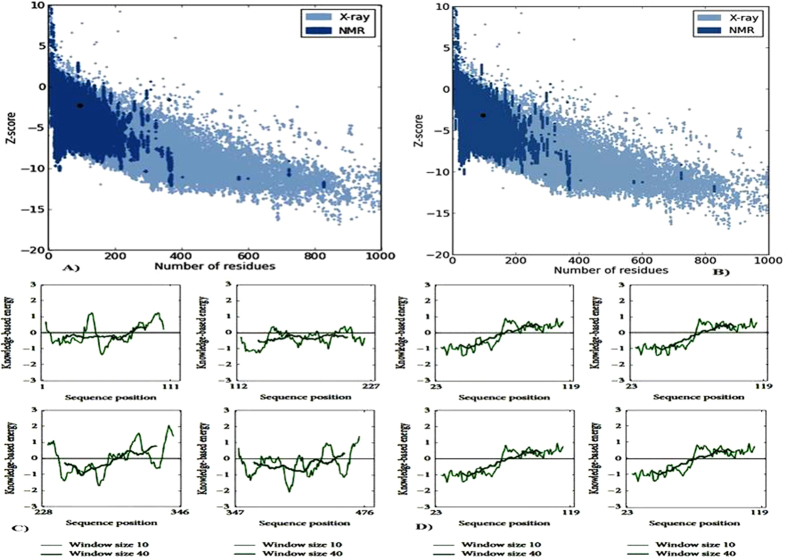
ProSA analyses of a structural model programmer of a protein structures plot check 3D for potential errors each repeat was examined separately. (**A**) ProSA black dot representation of for modelled LCC model fall in NMR based reliability with probable Z-score scale. (**B**) ProSA Z-scores for the template structure (KcsA) is similar like LCC model and fall within NMR based reliability represented in black dot. (**C**) ProSA energy profiles for LCC model (four repeats) 1 to 111, 112 to 227, 228 to 346, 347 to 476. (**D**) ProSA energy profiles for template (KcsA) (four repeats) 23 to 119, 23 to 119, 23 to 119, 23 to 119 and negative scores indicate a high quality model.

To assess the stereo chemical quality and structural integrity of the model, RAMPAGE^13^, ERRAT^14^, ProSA^15^,^16^ and Verify3-D^17^ software were used. For assessment, these methods were also worn to assess the template structure, and then each replicate was examined discretely by means of ProSA. RAMPAGE is a consequence of RAPPER which generates a Ramachandran plot by data derivative by Lovell *et al*.^13^. It is suggested that it be worn for this principle in favorite to PROCHECK, which is based on much older data. The Ramachandran diagram plots *phi* versus *psi* dihedral angles for every residue in the protein. The diagram is divided into favored, allowed, and disallowed regions, whose contouring is based on density-dependent smoothing for 81234 non-Glycine, non-Proline residues with B < 30 from 500 high resolution protein structures. Regions are also defined for Glycine, Proline, and pre-Proline as shown in Fig. 1. ProSA-Protein structure analysis program is a diagnostic tool that is based on the statistical analysis of all available protein structures^16^. It is a technique extensively used to verify 3D models of protein structures for possible errors. The energy of the structure is evaluated by a distance-based pair perspective and a possible that captures the solvent disclosure of protein residues. From these energies, two distinctiveness is consequent and displayed: Z-score and a plot of residue energies. The Z-score indicates in general model quality and measures the variation of the total energy of the structure with reference to an energy allotment derived from indiscriminate conformations. The overall quality score considered by ProSA for a precise structure is displayed in a plot that shows the scores computed from all experimentally resolute protein chains presently existing in the Protein Data Bank (PDB). The Z-scores for the model and template are much closer to the middle region of scores observed fore experimentally determined protein structures in the PDB including the template structure. The energy plot indicates the local model quality by plotting energies as a purpose of amino acid sequence. In general, positive values correspond to problematic or erroneous parts of the model (Fig. 2).

ERRAT is a protein structure verification algorithm, that is, especially well-suited for differentiating between correctly and incorrectly determined regions of protein structures based on characteristic atomic interactions^14^. The program provides an “overall quality factor” value which is defined as the percentage of the protein for which the calculated error value falls below the 95% statistical rejection limit. The ERRAT overall quality factor of the model is given in Supporting Information (Figure S2). This is not unexpected since the model has longer loops than the template. This process provides a helpful tool for model construction, structure confirmation, and construction decisions about consistency. A more reliable discrimination of incorrect regions would likely be obtained by combining the present analysis with others (Figure S2). Verify3-D analyzes the compatibility of an atomic model (3-D) with its own amino acid sequence (1-D) and hence tests the accuracy of the model. This technique evaluates the suitability of a protein sequence in its present 3-D environment. It can be applied to assess the quality of a theoretical model or to examine the characteristics of an experimental structure^17^. Table 1 shows the percentage of residues that had an average score >0.2 and the Verify3-D assessment of the structure (pass, warning, or fail) for the model and template (Figure S2) shows the Verify3-D profile for the model structure. Residues with a score over 0.2 should be considered reliable and the sequences exhibiting lower scores are those of extracellular loops. Taken collectively, all of the above information indicates that the excellence of the model is analogous to that of the template.

### Ligand Preparation

The chemical structures of all the title compounds 3 (a-e) and 5 (a-e) were established by IR, 1H and 13C NMR, mass spectral data and elemental analyses and their data are presented in the experimental section. IR bands in the regions1720–1760 and 1340–1375 cm-1 are assigned to C = O and S = O stretching vibrations respectively, for 3(a-e) and 5(a-e). The 1H NMR spectra exhibited triplet at δ 5.80–5.72, due to carbamate N–H protons and sulfonamide –NH proton gave a triplet at δ 3.26–3.19. The proton signals of Ar-H appeared as a multiple in the region δ 7.62–6.96. 13CNMR chemical shifts were observed in the region δ 151.2–155.6 for -NH-C = O.

### Spectral Characterization

The infrared spectral data of 3 (a–e) and 5 (a–e) are given in experimental parts. All the compounds 3 (a–e) and 5 (a–e) exhibited C = O stretching frequencies in the region 1720–1760 cm^−1^. All the compounds 3 (a-e) and 5 (a-e) showed S = O characteristic infrared absorption band in the region 1340–1375 cm^−1^.

The proton NMR chemical shifts of carbamates and sulfonamide derivatives of amlodipine 3 (a-e) and 5 (a-e) are given in Supporting information (S). The ^1^H NMR spectra exhibited triplet at δ 5.80–5.72, due to carbamate N–H protons and sulfonamide –NH proton gave a triplet at δ 3.26–3.19.The proton signals of Ar-H appeared as a multiple in the region δ 7.62–6.96. The remaining protons of carbamate and sulfonamide compounds were observed in the expected region.

The ^13^C NMR spectral data of carbamate and sulfonamide derivatives of amlodipine 3 (a-e) and 5 (a-e) are presented in Supporting information (S). ^13^C NMR chemical shifts were observed in the region δ 151.2–155.6 for -NH-C = O. The remaining carbon signals of carbamate and sulfonamide derivatives were observed in the expected region.

The LC-MS data of compounds **3** (a-e) and **5** (a-e) are given in the Supplementary data. The elemental analysis of the representative title compounds 3 (a-e) and 5 (a-e) is presented in the Supporting information (S).

The synthesized calcium channel blocker (CCB) structures were built with standard bond length and angles using ChemSketch v.2.1 (Figure S4) and then performed energy minimized. The minimized compounds were used for docking study.

### Molecular docking

**Figure 3:**
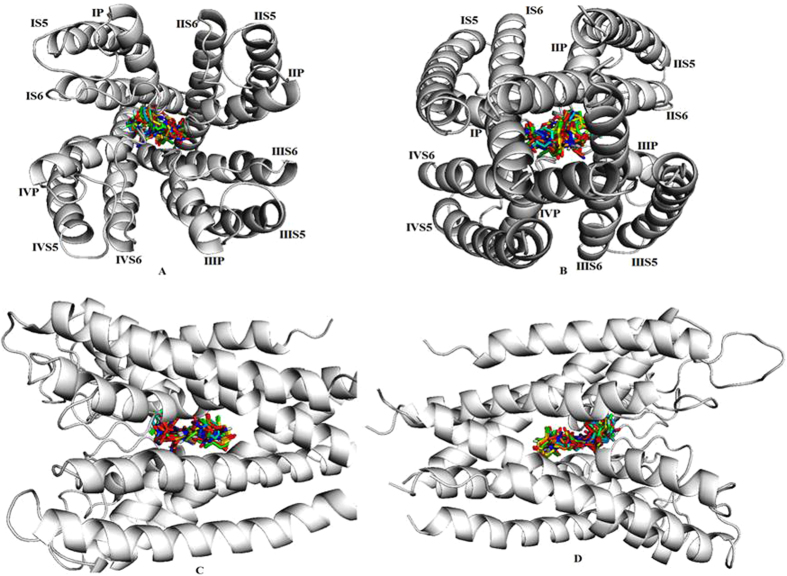
All ligands binding at core region of the active site of LCC model. (**A**) The bind mode of ligands and LCC model view of an interior poses view. (**B**) The bind mode of ligands and LCC model view of posterior poses view. (**C**) The bind mode of ligands and LCC model view of left side. (**D)** The bind mode of ligands and LCC model view of right side.

**Figure 4:**
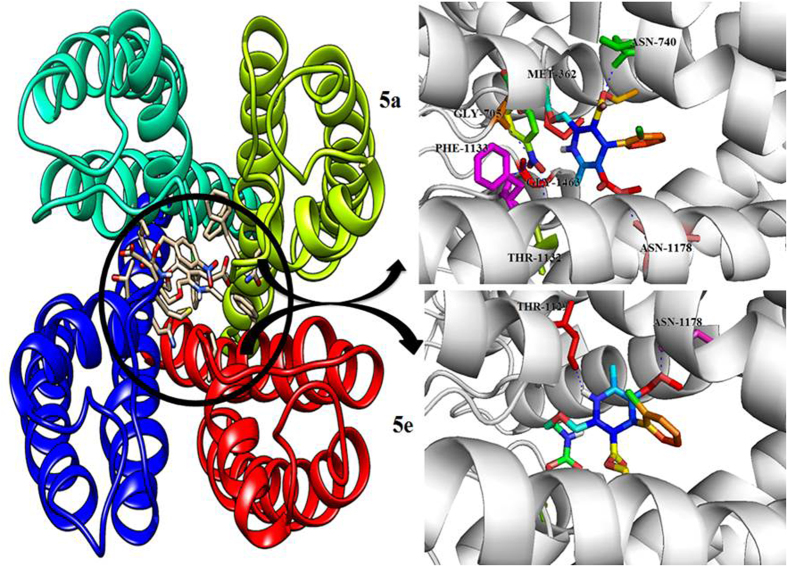
Active site of LCC model structure with four repeats regions with different color representations like Green, Red, Blue and Cyan where the docked interactions of **5a** and **5e** compounds displayed in dark circled ring in the core active site of LCC model. The novel dihydropyridine **5a** compound interactions are displayed as rainbow sticks, and key binding site residues are shown in green, yellow, red, pink and blue and hydrogen bonds as represented by dashed blue lines. The novel dihydropyridine **5e** compound interactions are displayed as rainbow sticks, and key binding site residues are shown in green, yellow, red, pink and blue and hydrogen bonds as represented by dashed blue lines.

In order to shed light on the molecular basis of the interactions between LCC and its ligands, docking simulations were undertaken on LCC model and synthesized compounds. Such calculations were conducted employing the automated docking program Auto Dock, which has proven to be really effective in reproducing the experimentally found posing of ligand in their binding site. As shown in Table 1, the predicted free energy of binding to all docked compounds and their surrounding amino acids. Docking of hits into the active site of the LCC model gave comparable binding solutions with the dihydropyridine ring fitting in the cleft formed by IIIS6, IIIS5, and IVS6 segments. The 3a compounds bound −8.1 binding energy with ASN-398 (IS6), THR-1129 (IIIP) and ASN-1178 (IIIS6) active site residues respectively. The 3b compounds bound −8.6 binding energy with ASN-740 (IIS6) active site residues respectively. The 3c compounds bound −9.8 binding energy with ASN-1178 (IIIS6), ASN-740 (IIS6) and ASN-398 (IS6) active site residues respectively. The 3d compounds bound −10.2 binding energy with THR-1132 (IIIP), PHE-1133 (IIIP), LEU-704 (IIP), ASN-740 (IIS6) and ASN-1178 (IIIS6) active site residues respectively. The 3e compounds bound −9.1 binding energy with THR-361 (IP), MET-362 (IP), THR-1129 (IIIP), THR-705 (IIP), ASN-1178 (IIIS6), ASN-740 (IIS6) active site residues respectively. The 5a compounds bound −12.1 binding energy with THR-1132 (IIIP), PHE-1133 (IIIP), GLY-1463 (IVP), GLY-705 (IIP), MET-362 (IP), ASN-740(IIS6) and ASN-1178 (IIIS6) active site residues respectively. The 5b compounds bound −8.9 binding energy with ASN-707 (IIP), GLY-705 (IIP), ASN-743 (IIS6) and THR-1129 (IIIP) active site residues respectively. The 5c compounds bound −9.6 binding energy with ASN-740 (IIS6) active site residues respectively. The 5d compounds bound −8.5 binding energy with ASN-1178 (IIIS6) active site residues respectively. The 5e compounds bound −11.0 binding energy with THR-1129 (IIIP) and ASN-1178 (IIIS6) active site residues respectively. All the synthesized compounds docked within the active site region of the LCC receptor pore (Fig. 3). The LCC model and docked compounds (5a and 5e) interactions were graphically represented in (Fig. 4). Thus the LCC model with Lipkind-Fozzard alignment and the pore bound ligands have a better ligand-receptor energy and higher number of contacts between CCBs (calcium channel blockers) and DHP (dihydropyridine) sensing residues. The structural analysis showed that most studied ligands fit into a binding pocket formed by Thr361, Met362, Asn398, Gly463, Gly705, Asn707, Asn740, Asn743, Thr1129, Thr1132, Phe1133, and Asn1178, and form hydrogen bond interactions.

### Molecular dynamics simulations

**Figure 5:**
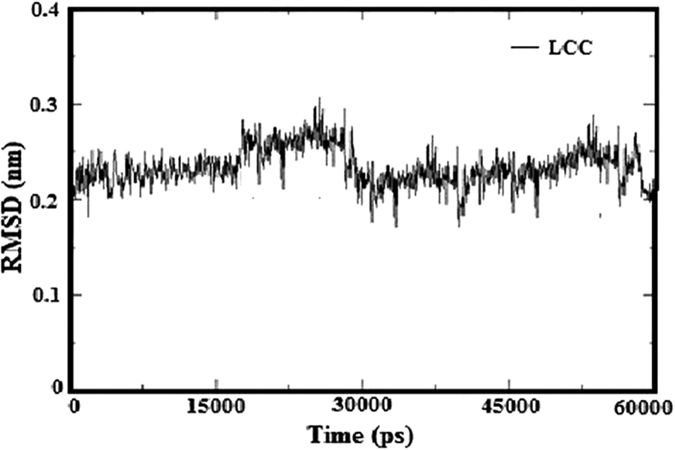
The root-mean-square deviation (RMSD) values of LCC model measure of the average distance between two atomic coordinates (usually Cα atoms of the entire protein) over 6 ns molecular dynamics simulations.

The homology model of LCC receptor was improved by molecular dynamics (MD) simulations with 6 ns of restrained positions. The stability of LCC model is analyzed by RMSD (root mean square deviation) (Fig. 5). The stability of the system was investigated using RMSD of Cα atoms of the protein as a function of the simulation time. The RMSD values of LCC rose to 0.27 nm and were relatively stable after 3 ns. During the first 3 ns the RMSD values are increased to 0.2 nm and stable. This simulation shows that the pore region of the improved open conformation is well behaved and rather stable after 3 ns.

**Figure 6:**
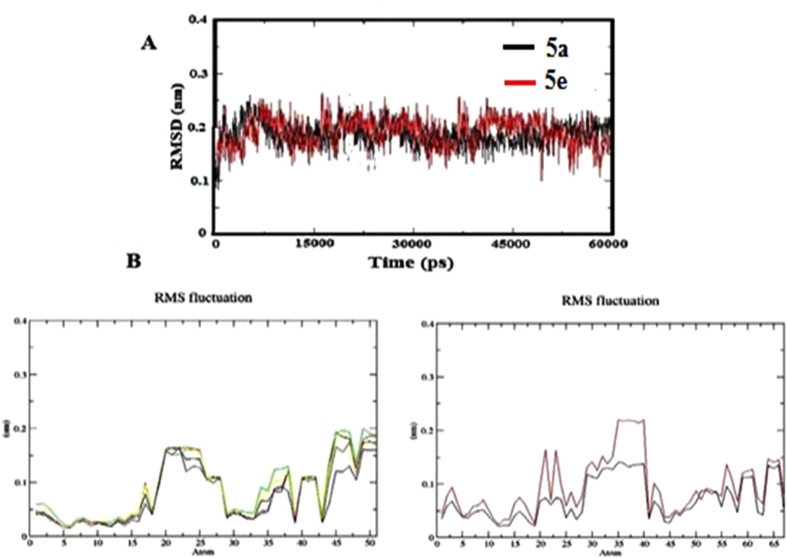
(**A**) The root-mean-square deviation (RMSD) of the LCC model and **5a** (black) complex and **5e** (red) complex. (**B**) The root-mean-square fluctuations (RMSF) measure of the average atomic mobility values of **5a** and **5e** compounds.

**Figure 7:**
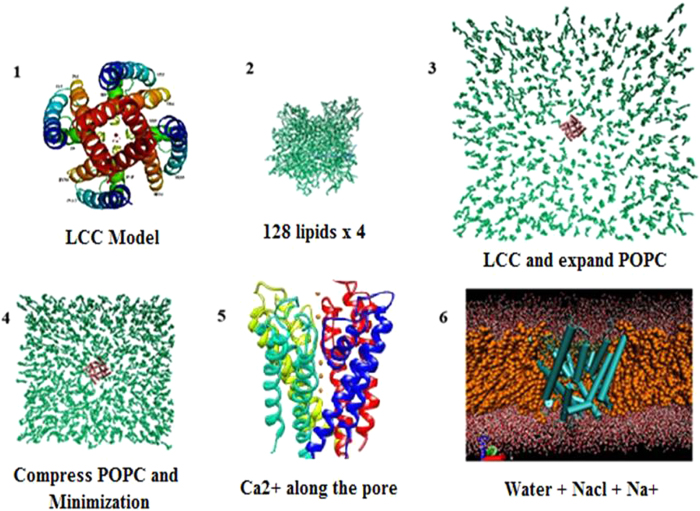
The setup of the molecular dynamics simulation of Cav1.2 channel in pure POPC. (**1**) Generate the topology file of LCC model conformation from homology modeling. (**2**) For POPC environment, replicate the starting configuration of a 128-lipid POPC bilayer in X and Y axis to create of a bilayer of 512 lipids. (**3**) Superimpose the LCC model with POPC and POPC/CHOL bilayer. (**4**) Compress and locate the channel in the center of bilayer and minimize the whole system. (**5**) Insert the 5 Ca^2+^ along the pore of LCC model. (**6**) Solvate water TIP3P, add NaCl neutralize system by adding Na^+^ ion.

The AD derivatives (**5a** and **5e**) were selected to gain insight into the structural and dynamic features of this ligand-receptor interaction. MD simulations of 5a and 5e were carried out initial orientations of the ligands. Analysis of the RMSD of the Cα atoms showed that all simulations reached an equilibrium after 4.5 ns. Additionally, the RMSD of 5a and 5e remained at small fluctuations after 1.5 ns of the simulation giving further confirmation of the stability of the simulation (Fig. 6). RMSF analyses of the ligands between 1 to 6 ns were also performed. Most of the flexible parts of 5a and 5e can be seen as peaks in the (Fig. 6). The hydrogen atoms are higher fluctuation than the other atoms. It is also indicated that the benzene ring and the other groups are rather rigid. The simulation setup is done by the following steps were shown in (Fig. 7).

### Biology

#### Intracellular Ca^2+^ measurements

The influence of novel synthesized compounds (3a-3e and 5a-5e) on intracellular calcium [Ca^2+^]i concentration was assessed in both the neuroblastoma SH-SY5Y cells (containing L- and N-type Ca^2+^ channels) and the rat aorta smooth muscle A7r5 cells (containing L-type Ca^2+^ channels). The known calcium channel blocker – Amlodipine was used as the positive control.

The evaluation of calcium antagonistic activities of novel synthesized compounds (3a-3e and 5a-5e) on SH-SY5Y and A7r7 cells. It described that compounds 5a, 5e showed best calcium antagonistic activity in both cells but these compounds higher activity in A7r7 cells. It indicating that compounds 5a and 5e predominantly targets the L-type calcium channels in vascular smooth muscles. The other compounds showed weak calcium antagonistic activity. In A7r7 cells, compounds 5a and 5e demonstrated at least 10 fold higher activities than that in SH-SY5Y cells (Table 2), indicating their potential vasodilation activity. The other compounds also showed good activity in A7r7 cells compared with SH-SY5Y cells. This experimental results also good acceptable with literature data, which demonstrated their calcium channel blocking activities.

#### Cadmium cytotoxicity studies

**Figure 8:**
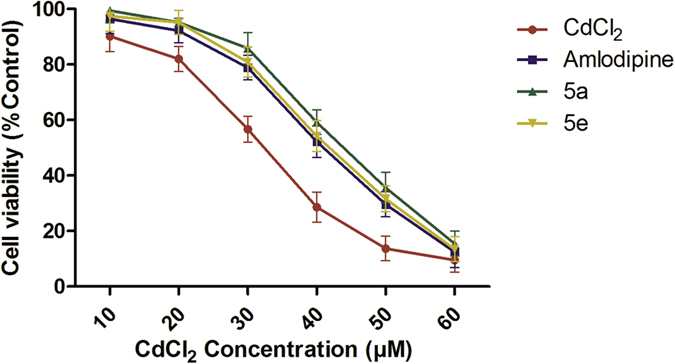
Cadmium toxicity effect of cell lines the effect of CdCl_2_ on 24 h cultures of isolated A7r5 cells after a 4 hr exposure represented curve with red color. The effect of a 1 hr Amlodipine (AD) pre-treatment on CdCl_2_ treated A7r5 cells represented curve with purple color. The effect of a 1 h **5a** compound pre-treatment on CdCl_2_ treated A7r5 cells represented curve with green color. The effect of a 1 hr **5e** compound pre-treatment onCdCl_2_ treated A7r5 cells represented curve with yellow color.

The CdCl_2_ induced cell death in A7r5 cells were determined in a dose-response manner following a 4 h dosing period with 10, 20, 30, 40, 50,60 μM concentrations (Fig. 8). The cell viability is a decrease in the concentration dependent of CdCl_2_. The following CdCl_2_ treatment with an LC_50_ of 30.2 ± 0.82 μM. The dihydropyridine such as AD, which is a potent Ca^2+^ channel blocker used as positive control. It was evaluated for its effect on CdCl_2_ toxicity. A 1 h pretreatment of A7r5 cells with 200 μM AD alone did not affect LDH leakage. A7r5 cells pretreated with 200 μM AD for 1 h exposure of different concentrations of CdCl_2_ showed a cytotoxicity response curve (Fig. 8). AD pretreatment ablated the 75% loss of cell viability at 20 μM CdCl_2_ concentration. The cytotoxicity response curve to the right yield an LC_50_ of 25.5 ± 0.53 μM. The CdCl_2_ alone treatment, the ratio of LC_50_ is 2.0 fold decrease in cell lethality in the presence of AD.

We also evaluated the synthesized novel calcium channel blockers compounds 5a and 5e effects on CdCl_2_ toxicity. A7r5 cells pretreated with 200 μM compounds 5a and 5e, for 1 h exposure of different concentrations of CdCl_2_ showed a cytotoxicity response curve (Fig. 8). The cytotoxicity curve to the right yield an LC_50_ is 2.5 and 2.3 fold decrease in cell lethality in the presence of compounds 5a and 5e respectively. The present study indicates that Cd uptake in smooth muscle cells through receptor-mediated Ca^2+^ channels.

The present work has demonstrated that synthesis, calcium channel blocking activity, homology modeling, docking, and molecular dynamic simulation studies for novel dihydropyridine derivatives, 10 of which were synthesized. Our molecules exhibited strong binding affinity with the LCC receptor during the molecular docking process. Molecular dynamic simulations, calcium channel blocking activity and cytotoxicity studies have been used as tools for selecting active drug molecules from a large volume of prospective compounds. We suggest these results demonstrate the ability of these procedures to accurately predict active drug candidates. The highest calcium channel blocking activity, was shown by compounds 5a and 5e. This activity, in smooth muscle cell line A7r5, was considerably higher than that in neuroblastoma cell lines SH-SY5Y, suggesting that these compounds are predominantly targeting the L-type calcium channels. These compounds possessed best calcium channel blocking activity if compared to 3a, 3b, 3c, 3d, 3e, 5b, 5c, and 5d. Compounds 5a and 5e at concentrations close to those of others demonstrating L-type calcium channel blocking activity did not affect mitochondrial functioning. These compounds can be considered as safe agents for further chemical modifications and studies in animal models of cardiovascular or neurological diseases.

## Material and Methods

### Homology modelling

The construction of the transmembrane region of the model was achieved by the employment of the modeller v 9.13. The structural model of the human LCC was built using the recently reported 3.20 Å crystal structure of KcsA^6^ (PDB entry code 1BL8) as a structural template. The sequence of the human LCC pore region alpha-1c subunit (Cav1.2, CAC1C_HUMAN) was retrieved from the SWISS-PROT database as well as aligned. The protocol used to develop the LCC model is divided into three phases: sequence alignment, model building, and model assessment.

### Sequence Alignment

The model was constructed using amino acid sequence of voltage-dependent LCC subunit alpha-1C (CAC1C HUMAN Q13936) obtained from UniProtKB/Swiss-Prot sequence database (http://www.uniprot.org/unipro/Q13936). Coordinates of potassium channel KcsA atoms in their closed conformation were downloaded from the Protein Data Bank (PDB ID: 1BL8). Amino acid sequences of S5, S6, and P-loops in between for the four repeats (I–IV) (271–405, 654–753, 1052–1185 and 1411–1524, respectively) were used for sequence alignment with the amino acid sequence of KcsA as proposed by Zhorov *et al*.^8^, shown in Figure S1 (Supporting Information (S)). In order to favor valid superimposition of the residues, the sequence of each repeat was ordered as S5, S6, and P-loop, allowing for an additional supple check of the results and easier corrections. The amino acid sequence of repeats I and III, has a long extracellular loop which would decrease the quality of the generated model, so amino acid sequences were excluded from repeats I and III, respectively with loop modelling process.

### Construction of the LCC Model

The modelling procedure consisted of two steps: model constructed from the template and refinement of loops. The above mentioned, described sequence alignment file was worn as enter in the modeller 9.15v program^18^ with the high resolution NMR structure of the potassium channel KcsA available in the RSCB Protein Data Bank (PDB ID: 1BL8) as a template for the 3D structure. The modeller Loop refinement-DOPE-Loop method^6^,^19^ was used for initial refinement of the loop conformation after model generation. The model side-chain conformation was optimized based on systematic searching of side-chain conformation and CHARMM energy minimization using the Chi Rotor algorithm^12^.

### Model assessment

The homology modelling (HM) phase was followed by the model evaluation phase. The stereo chemical quality and structural integrity of the model were tested by RAMPAGE, ERRAT, MolProbity, ProSA, and Verify3D tools and target–template superimposition by PyMol^20^ (Figure S3).

### Active site Identification

The active site of LCC model was identified using a CASTp server (Computer Atlas of Surface Topology of protein)^21^. The program, CASTp, for mechanically locating and measuring protein pockets and cavities, is based on accurate computational geometry methods, together with alpha shape and distinct flow theory.

### Ligand Preparations

Chemicals were bought from Sigma - Aldrich, Merck and Lancaster, and were utilized all things considered without further cleaning. All the solvents utilized for spectroscopic and other physical studies were reagent review and were further purged by literature methods. IR spectra were recorded as KBr pellets on a Perkin-Elmer 283 units. ^1^H and ^13^C NMR spectra were recorded on a Bruker 400 MHz NMR spectrometer working at 400 MHz for 1H, 100 MHz for 13C, they were recorded in CDCl3 and referenced to TMS (1H and 13C) and LC-MS spectra were recorded on a Jeol SX 102 DA/600 Mass spectrometer. Natural investigations were performed on a (Thermo Finnigan Instrument) at The University of Hyderabad, Hyderabad.

### Synthesis

Synthesis of 3-ethyl- 5-methyl- 2-(2-aminoethoxy)-4-(2-chlorophenyl)-1,4-dihydropyridine-3,5-dicarboxylate substituted carbamates 3 (a-e) and sulfonamide derivatives 5 (a-e) were achieved in two-steps. In the first step, free base of amlodipine (1) was obtained from the amlodipine besylate by treating with N, N-dimethyl piperazine (DMP) as a base in THF at 50–55 °C. The byproduct salt was removed from the reaction mixture by filtration. Subsequently, free base was reacted with various chloroformates 2 (a-e) and sulfonyl chlorides 4 (a-e) in the presence of N, N-dimethyl piperazine at 40–50 °C to obtain the title compounds 3 (a-e) and 5 (a-e) in 3–4 hr. Purity of the products and completion of the reaction was monitored by TLC using ethyl acetate: hexane (3:2). After completion of the reaction, DMP. Where the HCl was separated by filtration and the solvent was removed in a rota-evaporator and the residue were purified by column chromatography on silica gel (100–200 mesh) using hexane: ethyl acetate (2:1) as an eluent. The resulting title compounds 3 (a-e) and 5 (a-e) were obtained in high yields (68–80%).

The synthesized calcium channel blocker (CCB) structures were built with standard bond length and angles using ChemSketch v.2.1 and then performed energy minimized. The minimized compounds were used for docking study.

### Docking studies of Dihydropyridine (DHP) Amlodipine and its derivatives to the LCC model

Docking is a computational method which predicts the preferred orientation of one molecule to a second when bound to each other to form a stable complex. Docking has been widely used to suggest the binding modes of protein inhibitors. Most docking algorithms are able to generate a large number of possible structures, thus they also require a means to score each structure to identify those that of greatest interest. Docking was performed using Auto Dock in PyRx Virtual Screening tool^22^,^23^.

Ten synthesized compounds were docked into the active site of the refined LCC model. The Lamarkian genetic algorithm was used as number of individual population (150), max number of energy evaluation (25000000), max number of generation (27000)^24^, Gene mutation rate (0.02), crossover rate (0.8), Cauchy beta (1.0) and GA window size (10.0). The grid was set whole protein due to the multi binding pocket at X = 3.42, Y = −56.23, Z = 98.32 and dimension Å) at X = 89.92, Y = 98.56, Z = 98.32 and exhaustiveness 8. The pose for a given ligands identified on the basis of highest binding energy. Only ligand flexibility was taken into account and the proteins were considered to be rigid bodies. The resulting complexes were clustered according to their root mean square deviation (RMSD) values and binding energies, which were calculated using the Auto Dock scoring function. Further characterization via molecular dynamics (MD) simulations was conducted using complexes that were selected according to their binding energy values and the interactions made with the surrounding residues. The PyMol molecular viewer (http://www.pymol.org/) was employed to analyse the docked structures.

### Molecular dynamic simulations

Molecular Dynamic Simulation (MD) is a theoretical and computational method based on solving the Newton’s equation of motion. This method is used to mimic the behaviour of the system as a function of time. MD provides a basis for a more complete understanding of biological systems and aids in the interpretation of experiments concerned with their properties.

In a molecular dynamics simulation, the trajectory of the molecules and atoms for choosing the potential function U (r_1_, ….., r_N_) of the position of the nuclei represent the potential energy of the system when the atoms are arranged in specific configuration. The potential energy is usually constructed from the relative positions of the atoms with respect to each other. Forces are derived as the gradients of the potential with respect to the atomic displacement as shown in below formula.


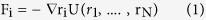


This form implies the presence of a conservation law of the total energy E = K + V, where K is the instantaneous kinetic energy. The translational motion of spherical molecules is caused by a force exerted by some external agent. The motion and the applied force are explicitly interpreted by Newtonian. Newton’s equation of motion of a particle system is written in a set of coupled second order differential equation in time. The functional form is a sum of terms:


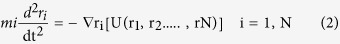


where *m* is the mass of the molecule, is a vector that locates the atoms with respect to a set of coordinate axes^25^.

All simulations were performed with Gromacs software version 4.0.7^26^ using the 43a1 force-field^27^. Both closed and open channels were embedded in a 1-palmitoyl-2-oeleoyl phosphatidylcholine (POPC) and POPC/CHOL lipid bilayer. The starting configuration of a 128-lipid POPC bilayer was obtained from the end of a 1.6 ns simulation performed by Kandt *et al*.^28^ (http://www.moose.bio.ucalgary.ca) and replicated four times to create a bilayer of 512 lipids. The INFLATEGRO^28^ tool was used to embed the channels in a POPC lipid bilayer. The POPC/CHOL lipid bilayer was generated by the following procedure: A cylindrical hole was introduced to implant the protein by removing a minimal number of overlapping POPC/CHOL lipids and compressing the bilayer using Gromacs tools. The system was solvated with around 25,339 SPC waters and included 0.1 mol/liter concentration of sodium chloride (NaCl). 5 Ca^2+^ ions were placed along the pore. The final POPC systems contained 502 POPC and 147 NaCl molecules. There were 537 POPC, 176 CHOL and 135 NaCl molecules in the closed channel POPC/CHOL system. The open channel POPC/CHOL system contained 525 POPC, 176 CHOL and 115 NaCl molecules. Na^+^ ions were added randomly within the solvent to neutralize the systems. The system was energy minimized with the steepest descent algorithm, followed by positional restrained MD for 2 ns. Subsequently, 10 ns of unrestrained MD simulation was carried out using the NPT ensemble. All simulations were repeated 5 times. The Nose-Hoover thermostat^29^ with a coupling constant of 0.1 ps to a temperature bath of 300 K was used. Pressure was held constant using a semi-isotropic Parrinello-Rahman barostat algorithm^30^ with a coupling constant of 1 ps. Electrostatic interactions were calculated explicitly at a distance smaller than 1 nm, and long-range electrostatic interactions were calculated at every step by particle-mesh Ewald summation^31^. Lennard-Jones interactions were calculated with a cutoff of 1 nm. Bond lengths were constrained using the LINCS algorithm Lennard-Jones interactions were calculated with a cut-off of 1 nm. Bond lengths were constrained using the LINCS algorithm^32^. Subsequent analyses of the MD simulation data were performed using the tools available within GROMACS.

### Molecular Dynamics simulation studies of the LCC model with 5a and 5e compounds

The simulation setup of all ligands with LCC model is the same procedure as following. Molecular dynamics simulations have been carried out for the LCC model embedded in DOPC^33^ lipid bilayer. These simulations were performed with the Gromacs software version 4.6.7 using the Amber-03 force-field. The topology of ligands was generated with antechamber. The ligand charges were taken from the quantum chemical calculation (Gaussian 03) with the Hartree - Fock 3–21 G basis set. The TIP3P water and 4 Ca^2+^ ions were placed along the z-axis. Cl- ions were added randomly within the solvent to neutralize the system.

The system was energy minimized with the steepest descent algorithm, followed by a positional restrained MD for 2 ns. Subsequently, 6 ns of unrestrained MD simulation was carried out using the NVT ensemble. The V-rescale thermostat and the Parrinello-Rahman barostat algorithms were used. Electrostatic interactions were calculated explicitly at a distance smaller than 1 nm, and long-range electrostatic interactions were calculated at every step by particle-mesh Ewald summation. Lennard-Jones interactions were calculated with a cut-off of 1 nm. All bonds were constrained by using the LINCS algorithm, allowing for an integration time step of 2 fs. The simulation temperature was kept constant by weakly (τ = 0.1 ps) coupling the lipids, protein, and solvent (water + counter ions) separately to a temperature bath of 300 K. The pressure was kept constant by weak coupling the system to a pressure bath of 1 bar with semi-isotropic pressure coupling.

### Biology

#### Intracellular Ca^2+^ measurements

The A7r5 (rat aorta smooth muscle cells) and SH-SY5Y (human neuroblastoma cell line) were obtained from the National Centre for Cell Science, Pune, India. The cells were grown at 37.5 °C in a humidified atmosphere with 5% CO_2_/95% air in Dulbecco’s modified Eagle medium (DMEM), restraining 2 mM of glutamine and supplemented with 10% fetal bovine serum. Once a week the cells were passaged using 0.25% trypsin, 0.53 mM EDTA (Ethylene diamine tetra acetic acid) solution and grown in 75 mm^2^ plastic culture flasks until confluence.

In the experiment, SH-SY5Y cells were plated in 96-well plates at a density of 30,000 cells per well and incubated for 24 h. A7r5 cells were seeded into 96-well plates at a density of 10,000 cells per well and incubated for 72 h. the intracellular [Ca^2+^]i concentration changes were studied using the Fluo-4 NW Calcium Assay Kit (Invitrogen, Sweden), as per the manufacturer’s instructions. Both SH-SY5Y and A7r5 cells were loaded with Fura-4NW for 45 min. Then, the cells were pre-incubated in the dark for 15 min with the tested compounds at concentrations from 1.0 to 100 mM. The compounds were dissolved in distilled water. Application of carbachol (100 nM) to Fura-4 loaded SH-SY5Y cells was used to stimulate changes in the intracellular [Ca^2+^]i concentration, whereas A7r5 cells were treated with 1.5 mM CaCl_2_ and KCl (50 mM) for 5 min to induce an increase in the [Ca^2+^] ion concentration. The intracellular [Ca^2+^] ion concentration was measured using the fluorescence spectrophotometer’s (Thermo Ascient, Hyderabad) settings appropriate for an excitation at 494 nm and an emission at 516 nm. Amlodipine was used as the positive control. IC_50_ values as mean ± SD were calculated using Graph Pad Prism 5.0 software.

#### Cadmium cytotoxicity studies

The A7r5 cell line was obtained from National Center for Cell Sciences, Pune, India. The cells were grown in Dulbecco’s Modified Eagles Medium (DMEM) supplemented with 5% fetal bovine serum at 37 °C in an atmosphere of 90% air/10% CO_2_. For determinations of cell viability, A7r5 cells were removed from stock flasks by mild digestion with trypsin-EDTA solution and the stock cultures were developed in 25 cm^2^ culture flasks and all trials were done in 96 micro titer plates. After plating, the cells were allowed to grow to confluence for 7–10 days before use.

The rat aorta smooth muscle A7r5 cells received one of the four treatments following overnight incubation. The first group received CdCl_2_ at 1, 10, 20, 30, 40, 50 μM prepared in complete culture for 4 h incubation. The second group received pretreatment with 200 μM AD for 1 h followed by the addition of 1, 10, 20, 30, 40, 50 μM CdCl_2_ for 4 h incubation. The third group received pretreatment with 200 μM compound 5a for 1 h followed by the addition of 1, 10, 20, 30, 40, 50 μM CdCl_2_ for 4 h incubation. The fourth group received pretreatment with 200 μM compound 5a for 1 h followed by the addition of 1, 10, 20, 30, 40, 50 μM CdCl_2_ for 4 h incubation. The CdCl_2_ different concentrations used in this test were chosen based on preliminary studies to identify concentrations which ranged from producing no effects to cytolethality. A short term exposure (4 h) was chosen to evaluate the effects of metallothionein induction^34^. The cell viability was assessed by measurement of (Lactate Dehydrogenase) LDH released into the medium^35^. LDH release was expressed as a percent of the maximum after lysis of cells with TritonX-100.

### Statistical analysis

The experiments were performed using three independent experiments. The values are expressed as means ± SE. Means were compared using one-way ANOVA for multiple- comparisons, followed by Turkey’s test. Statistical significance was set at P < 0.05.

## Additional Information

**How to cite this article**: Saddala, M. S. *et al*. Novel 1, 4-dihydropyridines for L-type calcium channel as antagonists for cadmium toxicity. *Sci. Rep.* **7**, 45211; doi: 10.1038/srep45211 (2017).

**Publisher's note:** Springer Nature remains neutral with regard to jurisdictional claims in published maps and institutional affiliations.

## Supplementary Material

Supplementary Information

## Figures and Tables

**Table 1:** Result of Amlodipine derivatives docking with LCC.

S.No.	Compound	BE (kcal/mol)	Surrounding Residues
1.	3a	−8.1	ASN-398 (**IS6**), THR-1129 (**IIIP**), ASN-1178 (**IIIS6**)
2.	3b	−8.6	ASN-740 (**IIS6**)
3.	**3c**	**−9.8**	ASN-1178 (**IIIS6**), ASN-740 (**IIS6**), ASN-398 (**IS6**)
4.	**3d**	**−10.2**	THR-1132 (**IIIP**), PHE-1133 (**IIIP**), LEU-704 (**IIP**), ASN-740 (**IIS6**), ASN-1178 (**IIIS6**)
5.	3e	−9.1	THR-361 (**IP**), MET-362 (**IP**), THR-1129 (**IIIP**), THR-705 (**IIP**), ASN-1178 (**IIIS6**), ASN-740 (**IIS6**)
6.	**5a**	**−12.0**	THR-1132 (**IIIP**), PHE-1133 (**IIIP**), GLY-1463 (**IVP**), GLY-705 (**IIP**), MET-362 (**IP**), ASN-740(**IIS6**), ASN-1178 (**IIIS6**)
7.	5b	−8.9	ASN-707 (**IIP**), GLY-705 (**IIP**), ASN-743 (**IIS6**), THR-1129 (**IIIP**)
8.	**5c**	**−9.6**	ASN-740 (**IIS6**)
9.	5d	−8.5	ASN-1178 (**IIIS6**)
10.	**5e**	**−11.0**	THR-1129 (**IIIP**), ASN-1178 (**IIIS6**)
11	Amlodipine	−8.4	ILE-360 (**IP**), SER-393 (**IS6**), ASN-1178 (**IIIS6**), ASN-740 (**IIS6**)

**Table 2:** Calcium overload-preventing activity (IC_50_) of novel synthesized compounds in A7r5 and SH-SY5Y cells.

S.No.	Compound	Cell lines (IC_50_)	
		A7r5	SH-SY5Y
1.	3a	4.81 ± 0.41	15 ± 0.28
2.	3b	7.42. ± 0.49	35 ± 0.24
3.	3c	7.02 ± 0.82	32 ± 0.45
4.	3d	4.25 ± 0.25	14 ± 0.39
5.	3e	8.35 ± 0.52	40 ± 0.43
6.	5a	0.18 ± 0.02*	8 ± 0.23*
7.	5b	0.52 ± 0.48	20 ± 0.52
8.	5c	0.43 ± 0.52	15 ± 0.53
9.	5d	0.59 ± 0.61	21 ± 0.49
10.	5e	0.25 ± 0.63*	10 ± 0.18*
11	Amlodipine	0.45 ± 0.52*	11 ± 0.61*
