# Supplementary material for: Novel 1, 4-dihydropyridines for L-type calcium channel as antagonists for cadmium toxicity
Source: Sci Rep. 2017 Mar 27;7:45211. doi: 10.1038/srep45211 (PMC5366925; doi:10.1038/srep45211)
Supplement: Supplementary Information [file srep45211-s1.pdf]

Novel 1, 4-dihydropyridines for L-type calcium channel as antagonists for cadmium toxicity

Madhu Sudhana Saddala[1], Ramesh Kandimalla[2], Pradeepkiran Jangampalli Adi[1, 2], Sainath Sri Bhashyam[3,4]*, Usha Rani Asupatri[1*]

[1]Sri Venkateswara University, Tirupati – 517502, A.P., India.

Division of Bioinformatics, DBT- Bioinformatics Centre,

Department of Zoology,

[2]Garrison Institute on Aging, Texas Tech University of Health Science Centre, Lubbock, USA.

[3]CIMAR/CIIMAR, Centro Interdisciplinar de Investigação Marinha e Ambiental,

Universidade do Porto, Rua dos Bragas, 177, 4050-123 Porto, Portugal.

[4]Vikrama Simhapuri University, Nellore-524 003, Andhra Pradesh,

India.

Supporting Information:

```
KcsA
M1                   AGAATVLLVIVLLAGSYLA 47
CAC1C_HUMAN
IS5                  IALLVLFVIIYAIIGLELF 290
IIS5                 LLLLFLFIIIFSLLGMQLF 673
IIIS5                VIVTTLLQFMFACIGVQLF 1071
IVS5                 ALLIVMLFFIYAVIGMQVF 1430

KcsA
P                    ITYPRALWWSVETATTVGYGD 80
CAC1C_HUMAN
IP                   DNFAFAMLTVFQCITMEGWTD 367
IIP                  DNFPQSLLTVFQILTGEDWNS 710
IIIP                 DNVLAAMMALFTVSTFEGWPE 1138
IVP                  QTFPQAVLLLFRCATGEAWQE 1468


KcsA
M2                   WGRCVAVVVMVAGITSFGLVTAALAT 112
CAC1C_HUMAN
IS6                  WPWIYFVTLIIIGSFFVLNLVLGVLS 405
IIS6                 LVCIYFIILFICGNYILLNVFLAIAV 753
IIIS6 (MODEL A)      VEISIFFIIYIIIIAFFMMNIFVGFV 1185
IIIS6 (MODEL B)      EISIFFIIYIIIIAFFMMNIFVGFVI 1186
IVS6                 FAVFYFISFYMLCAFLIINLFVAVIM 1524
```

Figure S1: Pairwise alignment of CAC1C_HUMAN and KcsA sequences. The conserved key residues used to align the sequences are shown in red boxes. Residues reported to affect DHPs antagonist binding and underscored and highlighted in bold.

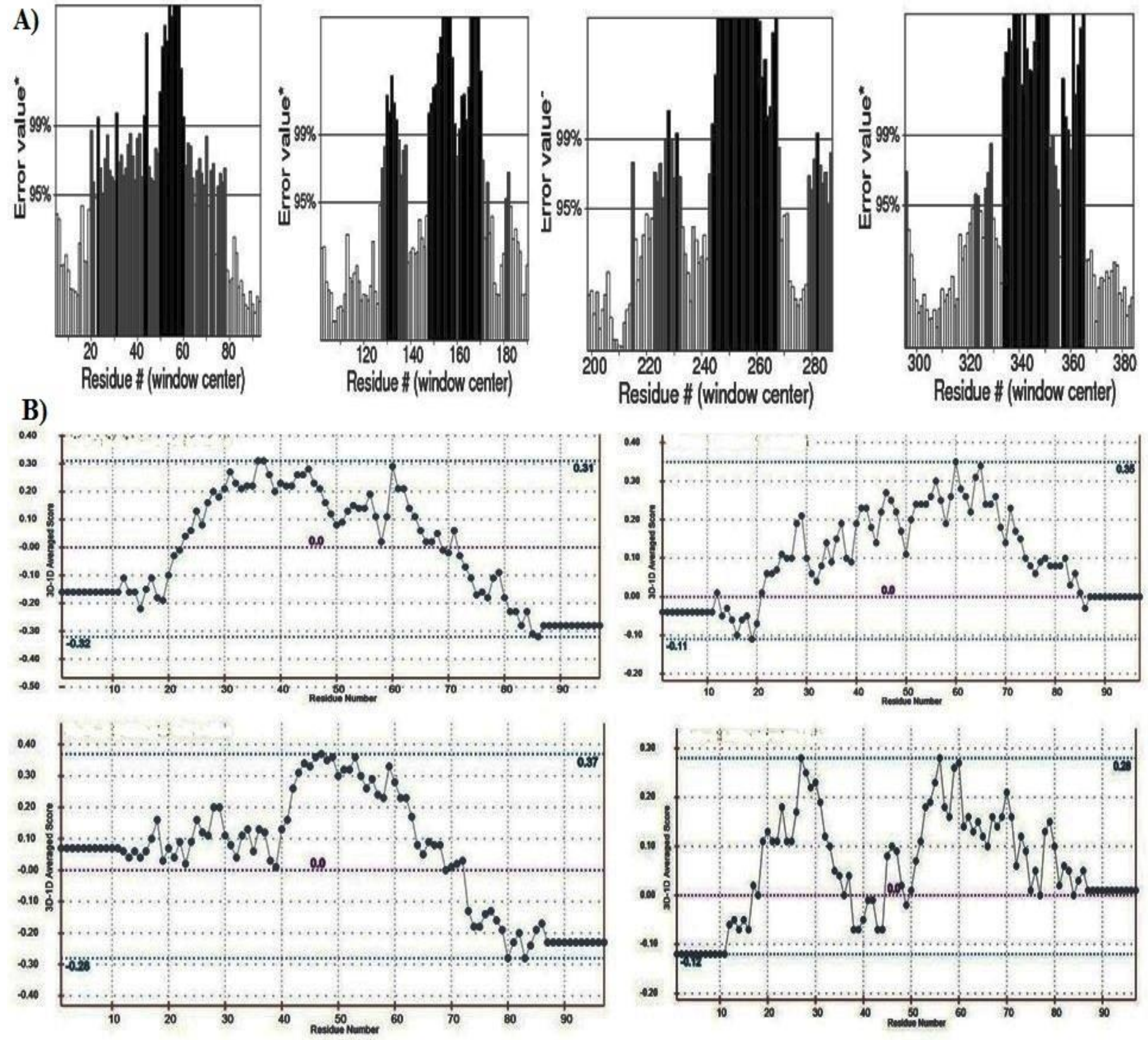

Figure S2: A) ERRAT score of the LCC model (four repeats) **B**) Verify3Dscore profile calculated for LCC model. Scores over 0.2 indicate a high quality model.

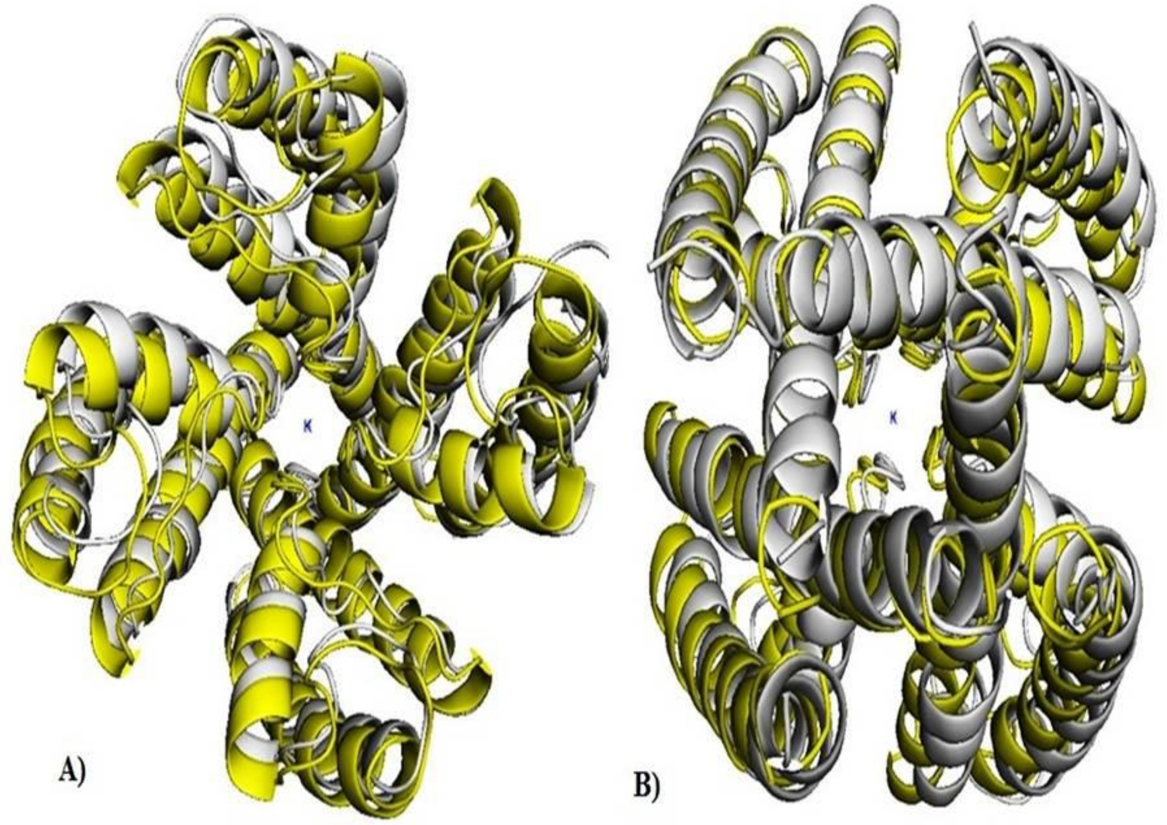

Figure S3: Superimposition of the LCC model (white) and KcsA (yellow) (PDB: 3BPM). (A) Open conformation, (B) Closed conformation.

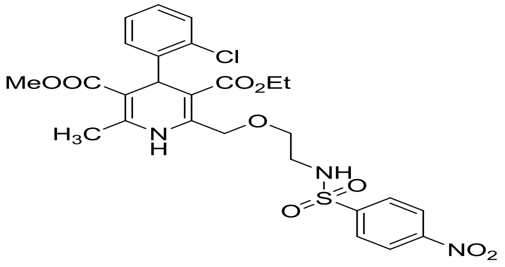

5a

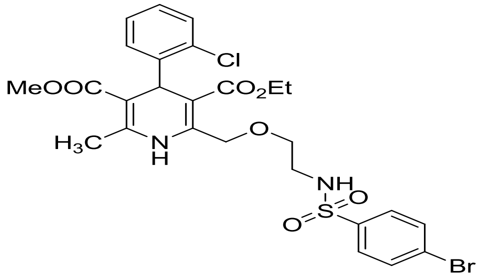

5b

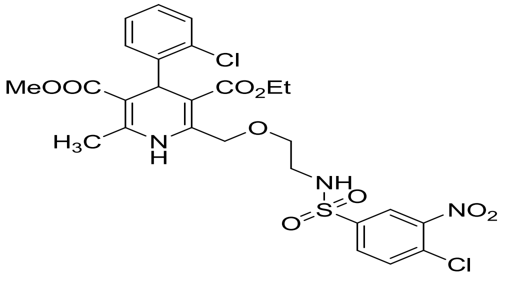

5c

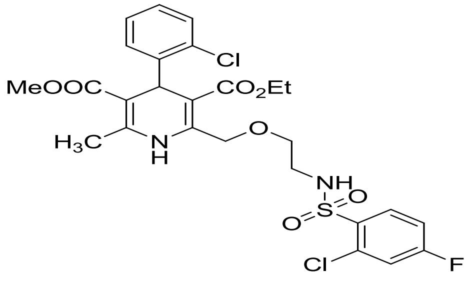

5d

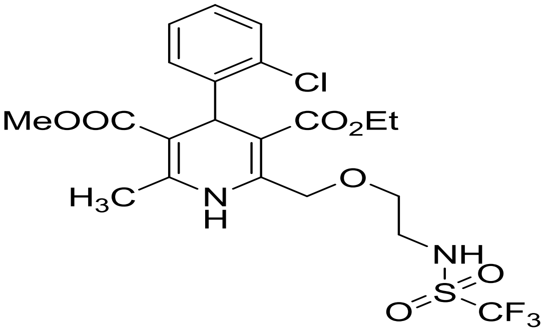

5e

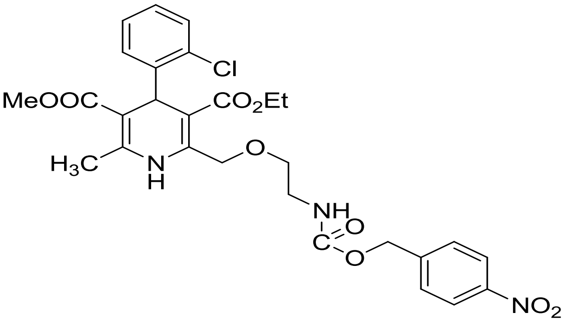

3a

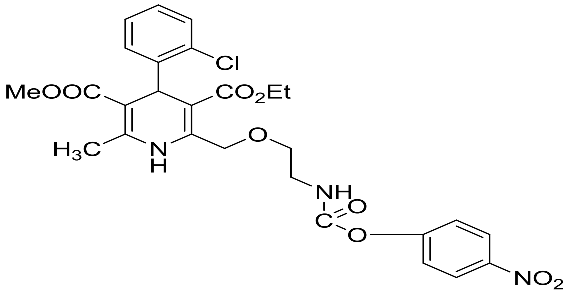

3b

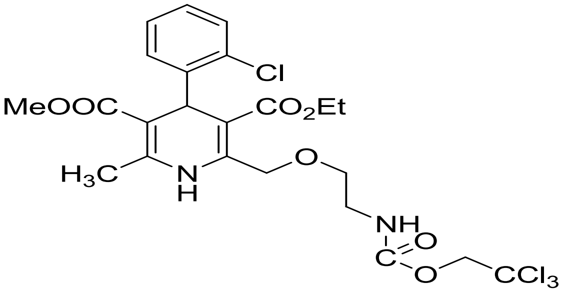

3c

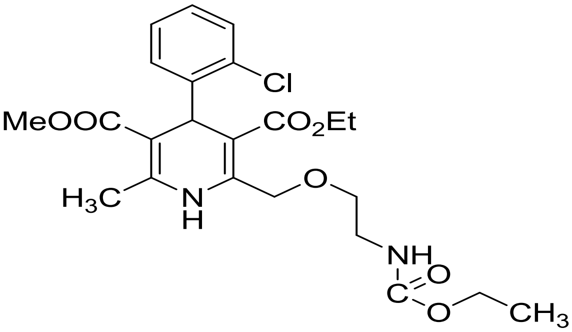

3d

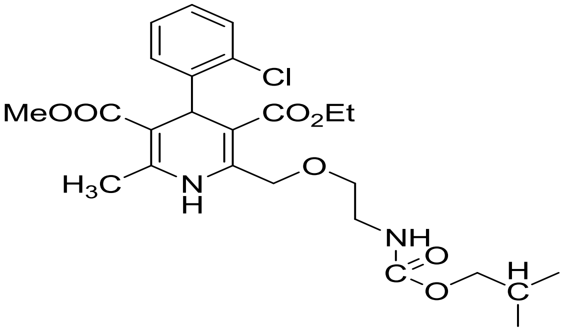

3e

Figure S4: The synthesized calcium channel blocker (CCB) structures were built with standard bond length and angles using ChemSketch.

Spectral data

3-Ethyl 5-methyl 4-(2-chlorophenyl)-2-((2-((4-nitrophenethoxy) carbonylamino) ethoxy) methyl)-1,4-dihydro pyridine-3,5-dicarboxylate (3a).

Yield 73%. mp 122-124 ^{o}C. υmax (KBr, cm-1): 3240 (-NH), 1748 (-C=O). 1H-NMR (400 MHz, CDCl3) δ (ppm): 8.24 (s, 1H, Ar-NH), 7.58-6.92 (m, 5H, Ar-H), 2.12 (3H, s, -CH3), 3.14 (3H, s, -OCH3), 4.16 (2H, q, -OCH2), 1.42 (3H, t, -CH3), 4.06 (2H, s, -OCH2), 3.72 (2H, t, -OCH2), 3.12 (2H, q, -CH2-NH), 5.74 (1H, t, -NH-C=O), 5.68 (2H, s, - CH2-Ar), 7.52 (2H, d, Ar'-H), 8.19 (2H, d, Ar'-H). 13C NMR (100 MHz, CDCl3) δ: 144.5 (C-2), 102.4 (C-3), 37.2 (C-4), 109.4 (C-5), 136.8 (C-6), 19.2 (C-7), 143.2 (C-8), 126.4 (C-9), 127.1 (C-10), 128.4 (C-11), 128.6 (C-12), 131.6 (C-13), 167.4 (C-14), 54.3 (C-16), 167.5 (C-17), 62.4 (C-19), 15.2 (C-20), 70.4 (C-21), 68.6 (C-23), 41.4 (C- 24), 155.4 (C-26), 65.6 (C-27), 141.2 (C-1'), 128.2 (C-2'), 124.2 (C-3'), 146.2 (C-4'). LC MS: m/z (%) 587.17 [100, M]+., 589 [65, M+2] +.. Anal.cald.for C28H30ClN3O9: C, 57.19; H, 5.14; N, 7.15; Found: C, 57.12, H, 5.09; N, 7.11.

3-Ethyl 5-methyl 4-(2-chlorophenyl)-2-((2-((4-nitrophenoxy)carbonylamino) ethoxy) methyl)-1,4-dihydro pyridine-3,5-dicarboxylate (3b).

Yield 72%. mp 132-134 ºC. υmax (KBr, cm-1): 3258 (-NH), 1720 (-C=O). 1H-NMR (400 MHz, CDCl3) δ (ppm): 8.16(s, 1H, Ar-NH), 7.54-6.96 (m, 5H, Ar-H), 2.16 (3H, s, -CH3), 3.18 (3H, s, -OCH3), 4.22 (2H, q, -OCH2), 1.54 (3H, t, -CH3), 4.15 (2H, s, -OCH2), 3.64 (2H, t, -OCH2), 3.24 (2H, q, -CH2-NH), 5.72 (1H, t, -NH-C=O), 7.58 (2H, d, Ar'-H), 8.26 (2H, d, Ar'-H). 13C NMR (100 MHz, CDCl3) δ: 144.2 (C-2), 101.5 (C-3), 36.6 (C-4), 109.4 (C-5), 136.2 (C-6), 19.4 (C-7), 142.6 (C-8), 127.2 (C-9), 126.9 (C-10), 128.5 (C-11), 129.2 (C-12), 131.4 (C-13), 166.2 (C- 14), 54.5 (C-16), 168.2 (C-17), 61.9 (C-19), 15.8 (C-20), 70.2 (C-21), 68.6 (C-23), 41.2 (C-24), 151.2 (C-26), 156.2 (C-1'), 122.2 (C-2'), 125.2 (C-3'), 144.5 (C-4'). LC MS: m/z (%) 573 [100, M]+., 575 [65, M+2] +.. Anal.cald.for C27H28ClN3O9: C, 56.50; H, 4.92; N, 7.32; Found: C, 56.48, H, 4.86; N, 7.28.

3-Ethyl 5-methyl 4-(2-chlorophenyl)-2-((2-((2,2,2-trichloroethoxy)carbonylamino) ethoxy)methyl)-1,4-dihydro pyridine-3,5-dicarboxylate (3c).

Yield 80%. mp 154-156 ºC. υmax (KBr, cm-1): 3236 (-NH), 1760 (-C=O). 1H-NMR (400 MHz, CDCl3) δ (ppm): 8.26(s, 1H, Ar-NH), 7.62-6.90 (m, 5H, Ar-H), 2.24 (3H, s, -CH3), 3.21 (3H, s, -OCH3), 4.26 (2H, q, -OCH2), 1.58 (3H, t,-CH3), 4.19 (2H, s, -OCH2), 3.60 (2H, t, -OCH2), 3.24 (2H, q, -CH2-NH), 5.78 (1H, t, -NH-C=O), 5.24 (2H, s, - OCH2). 13C NMR (100 MHz, CDCl3) δ: 144.6 (C-2), 102.4 (C-3), 36.8 (C-4), 109.8 (C-5), 136.6 (C-6), 19.2 (C-7), 142.8 (C-8), 127.5 (C-9), 127.2 (C-10), 128.6 (C-11), 129.6 (C-12), 131.6 (C-13), 166.6 (C-14), 54.8 (C-16), 168.8 (C-17), 61.2 (C-19), 15.6 (C-20), 70.5 (C-21), 66.4 (C-23), 41.5 (C-24), 153.4 (C-26), 71.2 (C-1'), 96.2 (C-2'). Anal.cald.for C23H26Cl4N2O7: C, 47.28; H, 4.49; N, 4.79; Found: C, 47.23, H, 4.45; N, 4.72.

3-Ethyl 5-methyl 4-(2-chlorophenyl)-2-((2-(ethoxy carbonylamino) ethoxy)methyl)-1,4-dihydropyridine-3,5- dicarboxylate (3d).

Yield 78%. mp 142-144 ºC. υmax (KBr, cm-1): 3230 (-NH), 1756 (-C=O). 1H-NMR (400 MHz, CDCl3) δ (ppm): 8.32(s, 1H, Ar-NH), 7.48-7.02 (m, 5H, Ar-H), 2.20 (3H, s, -CH3), 3.32 (3H, s, -OCH3), 4.21 (2H, q, -OCH2), 1.45 (3H, t, -CH3), 4.14 (2H, s, -OCH2), 3.54 (2H, t, -OCH2), 3.36 (2H, q, -CH2-NH), 5.78 (1H, t, -NH-C=O), 4.26 (2H, q, - OCH2), 1.42 (3H, t, -CH3); 13C NMR (100 MHz, CDCl3) δ: 144.2 (C-2), 103.2 (C-3), 36.4 (C-4), 109.6 (C-5), 136.4(C-6), 19.5 (C-7), 142.5 (C-8), 127.2 (C-9), 126.8 (C-10), 127.8 (C-11), 129.4 (C-12), 131.2 (C-13), 166.9 (C-14), 54.4 (C-16), 168.2 (C-17), 61.6 (C-19), 15.2 (C-20), 71.2 (C-21), 66.5 (C-23), 40.6 (C-24), 155.6 (C-26), 61.2 (C- 1'), 13.7 (C-2'). Anal.cald.for C23H29ClN2O7: C, 57.44; H, 6.08; N, 5.82; Found: C, 57.38, H, 6.02; N, 5. 78.

3-Ethyl 5-methyl 4-(2-chlorophenyl)-2-((2-(isobutoxycarbonylamino)ethoxy)methyl)-1,4-dihydropyridine-3,5-dicarboxylate (3e).

Yield 74%. mp 166-168 ºC. υmax (KBr, cm-1): 3256 (-NH), 1752 (-C=O). 1H-NMR (400 MHz, CDCl3) δ (ppm): 8.28(s, 1H, Ar-NH), 7.58-7.06 (m, 5H, Ar-H), 2.08 (3H, s, -CH3), 3.36 (3H, s, -OCH3), 4.18 (2H, q, -OCH2), 1.48 (3H, t, -CH3), 4.26 (2H, s, -OCH2), 3.62 (2H, t, -OCH2), 3.32 (2H, q, -CH2-NH), 5.80 (1H, t, -NH-C=O), 3.72 (2H, t, - OCH2), 1.92-1.88 (1H, m, -CH), 1.04 (6H, d, -(CH3)2). 13C NMR (100 MHz, CDCl3) δ: 145.2 (C-2), 102.5 (C-3),36.1 (C-4), 109.2 (C-5), 136.6 (C-6), 19.7 (C-7), 142.4 (C-8), 127.4 (C-9), 126.2 (C-10), 127.4 (C-11), 129.2 (C-12),131.4 (C-13), 166.3 (C-14), 54.2 (C-16), 167.4 (C-17), 61.2 (C-19), 15.6 (C-20), 71.4 (C-21), 66.5 (C-23), 40.6 (C-24), 152.8 (C-26), 71.2 (C-1'), 26.7 (C-2'), 20.1 (C-3'). LC MS: m/z (%) 508 [100, M]+., 410 [65, M+2] +..Anal.cald.for C25H33ClN2O7: C, 58.99; H, 6.53; N, 5.50; Found: C, 58.92, H, 6.48; N, 5.42.

3-Ethyl 5-methyl 4-(2-chlorophenyl)-2-((2-(4-nitrophenylsulfonamido)ethoxy)methyl)-1,4-dihydropyridine-3,5-dicarboxylate (5a).

Yield 75%. mp 142-144 ^{o}C. υmax (KBr, cm-1): 3262 (-NH), 1348 (-S=O). 1H-NMR (400 MHz, CDCl3) δ (ppm): 8.28(s, 1H, Ar-NH), 7.62-7.12 (m, 5H, Ar-H), 2.12 (3H, s, -CH3), 3.12 (3H, s, -OCH3), 4.25 (2H, q, -OCH2), 1.58 (3H, t,-CH3), 4.19 (2H, s, -OCH2), 3.52 (2H, t, -OCH2), 3.28 (2H, q, -CH2-NH), 3.22 (1H, t, -NH-SO2), 8.24 (2H, d, Ar'-H), 8.42 (2H, d, Ar'-H). 13C NMR (100 MHz, CDCl3) δ: 143.2 (C-2), 100.6 (C-3), 35.4 (C-4), 108.2 (C-5), 135.5(C-6), 18.8 (C-7), 141.8 (C-8), 126.4 (C-9), 125.2 (C-10), 127.2 (C-11), 128.5 (C-12), 130.2 (C-13), 165.4 (C-14),54.2 (C-16), 162.4 (C-17), 62.4 (C-19), 15.6 (C-20), 69.1 (C-21), 68.4 (C-23), 41.6 (C-25), 152.4 (C-1'), 128.2 (C-2'), 124.2 (C-3'), 150.6 (C-4'). LC MS: m/z (%) 593 [100, M]+., 595 [65, M+2] +.. Anal.cald.for C26H28ClN3O9S: C,52.57; H, 4.75; N, 7.07; Found: C, 52.52, H, 4.70; N, 7.01.

3-Ethyl 5-methyl 2-((2-(4-bromophenylsulfonamido)ethoxy)methyl)-4-(2-chlorophenyl)-1,4-dihydropyridine-3,5-dicarboxylate (5b).

Yield 76%. mp 156-158 ^{o}C. υmax (KBr, cm-1): 3315 (-NH), 1352 (-S=O). 1H-NMR (400 MHz, CDCl3) δ (ppm): 8.20(s, 1H, Ar-NH), 7.56-7.02 (m, 5H, Ar-H), 2.06 (3H, s, -CH3), 3.14 (3H, s, -OCH3), 4.22 (2H, q, -OCH2), 1.54 (3H,t, -CH3), 4.20 (2H, s, -OCH2), 3.56 (2H, t, -OCH2), 3.22 (2H, q, -CH2-NH), 3.26 (1H, t, -NH-SO2), 7.92 (2H, d, Ar'-H), 8.04 (2H, d, Ar'-H). 13C NMR (100 MHz, CDCl3) δ: 144.5 (C-2), 101.6 (C-3), 37.2 (C-4), 107.4 (C-5), 136.2(C-6), 18.6 (C-7), 142.4 (C-8), 126.8 (C-9), 124.3 (C-10), 128.4 (C-11), 128.2 (C-12), 131.5 (C-13), 166.5 (C-14),55.4 (C-16), 163.2 (C-17), 63.5 (C-19), 16.2 (C-20), 68.6 (C-21), 65.6 (C-23), 41.4 (C-25), 144.2 (C-1'), 129.4 (C-2'), 130.4 (C-3'), 128.2 (C-4'). LC MS: m/z (%) 626 [100, M]+., 628 [98, M+2] +.. Anal.cald.for C26H28BrClN2O7S:C, 49.73; H, 4.49; N, 4.46; Found: C, 49.68, H, 4.43; N, 4.42.

3-Ethyl 5-methyl 2-((2-(4-chloro-3-nitrophenylsulfonamido)ethoxy)methyl)-4-(2-chlorophenyl)-1,4-dihydropyridine-3,5-dicarboxylate (5c).

Yield 79%. mp 169-171 ^{o}C. υmax (KBr, cm-1): 3354 (-NH), 1375 (-S=O). 1H-NMR (400 MHz, CDCl3) δ (ppm): 8.32(s, 1H, Ar-NH), 7.56-6.92 (m, 5H, Ar-H), 2.14 (3H, s, -CH3), 3.15 (3H, s, -OCH3), 4.20 (2H, q, -OCH2), 1.52 (3H, t,-CH3), 4.26 (2H, s, -OCH2), 3.52 (2H, t, -OCH2), 3.28 (2H, q, -CH2-NH), 3.25 (1H, t, -NH-SO2), 8.06 (1H, d, Ar'-H), 8.24 (1H, d, Ar'-H), 8.62 (1H, s, Ar'-H). 13C NMR (100 MHz, CDCl3) δ: 145.2 (C-2), 102.4 (C-3), 38.6 (C-4),108.2 (C-5), 138.2 (C-6), 18.5 (C-7), 140.4 (C-8), 128.2 (C-9), 125.4 (C-10), 128.4 (C-11), 128.6 (C-12), 132.4 (C-13), 168.2 (C-14), 55.6 (C-16), 163.4 (C-17), 63.2 (C-19), 16.6 (C-20), 68.2 (C-21), 65.4 (C-23), 41.5 (C-25), 138.2(C-1'), 134.6 (C-2'), 130.4 (C-3'), 132.5 (C-4'), 146.2 (C-5'), 125.2 (C-6'). Anal.cald.for C26H27Cl2N3O9S: C, 49.69;H, 4.33; N, 6.69; Found: C, 49.64, H, 4.29; N, 6.62.

3-Ethyl 5-methyl 4-(2-chlorophenyl)-2-((2-(4-fluorophenylsulfonamido) ethoxy)methyl) -1,4-dihydropyridine-3,5-dicarboxylate (5d).

Yield 70%. mp 176-178 ^{o}C. υmax (KBr, cm-1): 3342 (-NH), 1366 (-S=O). 1H-NMR (400 MHz, CDCl3) δ (ppm): 1HNMR(CDCl3) δ (ppm): 8.28 (s, 1H, Ar-NH), 7.58-6.95 (m, 5H, Ar-H), 2.17 (3H, s, -CH3), 3.24 (3H, s, -OCH3),4.26 (2H, q, -OCH2), 1.62 (3H, t, -CH3), 4.24 (2H, s, -OCH2), 3.52 (2H, t, -OCH2), 3.26 (2H, q, -CH2-NH), 3.21(1H, t, -NH-SO2), 7.96 (2H, d, Ar'-H), 7.52 (2H, d, Ar'-H). 13C NMR (CDCl3) δ: 144.2 (C-2), 102.4 (C-3), 37.5 (C-4), 107.2 (C-5), 136.6 (C-6), 18.8 (C-7), 142.8 (C-8), 126.6 (C-9), 124.8 (C-10), 127.6 (C-11), 127.8 (C-12), 131.2(C-13), 166.2 (C-14), 56.2 (C-16), 164.4 (C-17), 63.2 (C-19), 16.5 (C-20), 68.2 (C-21), 65.4 (C-23), 41.3 (C-25),141.2 (C-1'), 130.8 (C-2'), 115.4 (C-3'), 166.5 (C-4'). Anal.cald.for C26H27Cl2FN2O7S: C, 51.92; H, 4.52; N, 4.66;Found: C, 51.88, H, 4.48; N, 4.61.

3-Ethyl 5-methyl 4-(2-chlorophenyl)-2-((2-(trifluoromethylsulfonamido) ethoxy)methyl) -1,4-dihydropyridine-3,5-dicarboxylate (5e).

Yield 68%. mp 181-183 ºC. υmax (KBr, cm-1): 3328 (-NH), 1372 (-S=O). 1H-NMR (400 MHz, $CDCl_3$) δ (ppm): 8.32(s, 1H, Ar-NH), 7.52-6.94 (m, 5H, Ar-H), 2.15 (3H, s, $-CH_3$), 3.38 (3H, s, $-OCH_3$), 4.42 (2H, q, $-OCH_2$), 1.68 (3H, t,$-CH_3$), 4.34 (2H, s, $-OCH_2$), 3.61 (2H, t, $-OCH_2$), 3.28 (2H, q, $-CH_2$-NH), 3.24 (1H, t, $-NH-SO_2$). 13C NMR(100MHz, $CDCl_3$) δ: 145.2 (C-2), 103.5 (C-3), 38.2 (C-4), 108.2 (C-5), 137.2 (C-6), 19.0 (C-7), 143.5 (C-8), 127.2(C-9), 125.2 (C-10), 128.2 (C-11), 129.2 (C-12), 134.4 (C-13), 167.2 (C-14), 57.2 (C-16), 166.4 (C-17), 64.4 (C-19),16.7 (C-20), 69.4 (C-21), 65.6 (C-23), 41.8 (C-25), 150.2 (C-1'). LC MS m/z (%) 540 [100, M]+., 542 [65, M+2] +..Anal.cald.for $C_{21}H_{24}ClF_3N_2O_7S$: C, 46.63; H, 4.47; N, 5.18; Found: C, 46.58, H, 4.42; N, 5.12.
